# Prevalence and factors associated with the use of alternative (folk) medicine practitioners in 8 countries of the former Soviet Union

**DOI:** 10.1186/1472-6882-13-83

**Published:** 2013-04-11

**Authors:** Andrew Stickley, Ai Koyanagi, Erica Richardson, Bayard Roberts, Dina Balabanova, Martin McKee

**Affiliations:** 1 European Centre on Health of Societies in Transition, London School of Hygiene and Tropical Medicine, 15-17 Tavistock Place, London WC1H 9SH, UK; 2 Stockholm Centre on Health of Societies in Transition (SCOHOST), Södertörn University, Huddinge 141 89, Sweden; 3 European Observatory on Health System and Policies, London School of Hygiene and Tropical Medicine, 15-17 Tavistock Place, London, WC1H 9SH, UK

**Keywords:** Alternative (folk) medicine, Former Soviet Union, Rural residence, Medical symptoms, Prevalence

## Abstract

**Background:**

Research suggests that since the collapse of the Soviet Union there has been a sharp growth in the use of complementary and alternative medicine (CAM) in some former Soviet countries. However, as yet, comparatively little is known about the use of CAM in the countries throughout this region. Against this background, the aim of the current study was to determine the prevalence of using alternative (folk) medicine practitioners in eight countries of the former Soviet Union (fSU) and to examine factors associated with their use.

**Methods:**

Data were obtained from the Living Conditions, Lifestyles and Health (LLH) survey undertaken in eight former Soviet countries (Armenia, Belarus, Georgia, Kazakhstan, Kyrgyzstan, Moldova, Russia and Ukraine) in 2001. In this nationally representative cross-sectional survey, 18428 respondents were asked about how they treated 10 symptoms, with options including the use of alternative (folk) medicine practitioners. Multivariate logistic regression analysis was used to determine the factors associated with the treatment of differing symptoms by such practitioners in these countries.

**Results:**

The prevalence of using an alternative (folk) medicine practitioner for symptom treatment varied widely between countries, ranging from 3.5% in Armenia to 25.0% in Kyrgyzstan. For nearly every symptom, respondents living in rural locations were more likely to use an alternative (folk) medicine practitioner than urban residents. Greater wealth was also associated with using these practitioners, while distrust of doctors played a role in the treatment of some symptoms.

**Conclusions:**

The widespread use of alternative (folk) medicine practitioners in some fSU countries and the growth of this form of health care provision in the post-Soviet period in conditions of variable licensing and regulation, highlights the urgent need for more research on this phenomenon and its potential effects on population health in the countries in this region.

## Background

Globally, the use of alternative and complementary medicine is widespread. In Africa, it has been estimated that up to 80% of the population uses ‘traditional medicine’ [1] while studies from high income Western countries such as the United States and Germany suggest that the prevalence of complementary and alternative medicine (CAM) use ranges between 5% and 62% [2]. Comparisons over time also suggest that its use may be increasing [3,4]. Indeed, surveys indicate that these treatments are used by all age groups [5,6] and for a wide variety of conditions, including non-communicable diseases, [7] psychological conditions such as anxiety and depression, [8,9] and life-threatening illnesses that include cancer and HIV infection [10,11].

Despite growing research on the patterns of use of CAM practitioners, there are still many gaps in our knowledge [12]. This is particularly true in Europe. Thus, although there is some evidence that CAM is used extensively in this region [13,14], the available data come from only a few countries, mainly in Western Europe [14]. This is an important research gap. Such treatments can involve sizable out-of-pocket expenditure [3,15] while in some instances they can be associated with side effects – which while mostly transient [10], may nevertheless entail serious outcomes in some cases [16].

This study begins to fill an important geographical gap in our knowledge by examining the patterns of use of alternative (folk) medicine practitioners in the countries of the former Soviet Union (fSU). Although the use of non-biomedical therapies has deep roots in this region [17], the Soviet regime institutionalised biomedicine and banned alternative practice in the USSR in 1923 [18]. Nevertheless, despite various forms of persecution (fines, imprisonment) non-biomedical practitioners continued to provide services throughout the Soviet period – principally in rural locations – that were either periodically unavailable (e.g. abortion) or inaccessible [19], while self-treatment with alternative folk remedies was also widespread [20] in an environment where shortages of conventional pharmaceutical medicines were commonplace [21,22]. Official attitudes to non-biomedical forms of treatment softened somewhat in the later Soviet period with the recognition of some forms of CAM as a speciality in 1977 [23] which stimulated a resurgence in alternative treatments in the 1980s [24].

Some evidence suggests that there has been a pronounced growth in differing forms of CAM in Eastern Europe in recent years. This has been facilitated not only by the removal of legal and social prohibitions following the collapse of the communist system [14] but also by a revival of traditional culture, a growth in out-of-pocket payments for conventional medical services [25-27], and in some former Soviet republics, the collapse of the earlier system offering universal coverage. This growth in CAM, which has also included a sharp increase in the number of practitioners [28], has occurred against a backdrop of differing regulatory environments within the former Soviet countries [14]. Even though alternative practitioners tend to deal with minor health problems [19], there is some suggestion that as a result of using CAM, patients may have delayed obtaining needed conventional treatment [19], while in Ukraine, it has been claimed that the uncontrolled spread of various healing practices may have started to impact detrimentally on population health in the post-Soviet period [28].

Given evidence that there are now large numbers of CAM practitioners operating in some former Soviet countries [17,28], many without formal training or connection with conventional health care [28], there is a need to know more about this phenomenon. However, there is little or no information about the use of any form of CAM in many countries in this region [14,29,30]. In an attempt to address this potentially important research gap, this study examines the extent to which alternative (folk) medicine practitioners are being used in eight former Soviet countries, for what symptoms and the factors associated with their use. Specifically, this will shed light on the extent to which these alternative practitioners are being consulted for minor illnesses, a question of particular relevance in some of our study countries such as Ukraine where they are forbidden to treat certain diseases (e.g. cancer, infectious diseases and severe mental disorders) [28].

## Methods

### Study population

The data come from the Living Conditions, Lifestyles and Health (LLH) study undertaken in eight former Soviet countries (Armenia, Belarus, Georgia, Kazakhstan, Kyrgyzstan, Moldova, Russia and Ukraine) in 2001. Details of the survey methodology have been presented elsewhere [31]. In brief, standardised methods were used for each country survey to obtain a representative sample of the population aged 18 years and above although several small regions were excluded in Georgia (South Ossetia), Moldova (Transnistria) and the Russian Federation (Chechnya) because of separatist movements or ongoing military conflict. Similarly, at the individual-level, those who were in the armed forces or prisoners were excluded as were the mentally incapacitated, institutionalised, hospitalised or homeless, or individuals who were intoxicated. The target sample was 2000 per country apart from Ukraine (2500) and Russia (4000) where there were larger samples to reflect their larger and more diverse populations.

Multi-stage random sampling with stratification by region and rural/urban settlement type was used. Within each primary sampling unit (about 50–200 per country), households were selected by random sampling from a household list (Armenia) or by standardised random route procedures (other countries). One respondent was obtained from each selected household (according to the nearest birthday). If there was no one at home after three visits (on different days and at different times), the next household on the route was visited. A pre-specified quota control was used in Belarus, Kazakhstan, Moldova and Ukraine (a combination of region, area, gender, age and/or education level), while a sampling repair procedure (based on area, gender, age and education) was employed in Georgia and Russia. Trained fieldworkers conducted face-to-face interviews in the respondents’ homes. Before being included in the study, respondents gave their verbal consent to participate. The questionnaire was developed and piloted in consultation with country representatives. All respondents were given the choice of answering either in Russian or their own national language except in Russia and Belarus where Russian was used. Re-interviews to assess the work of both the interviewers and the interviewers’ supervisors were undertaken as quality control procedures. Response rates varied between 71% and 88% among countries with the final study sample consisting of 18428 individuals. As regards the present study, after the exclusion of respondents missing information on any of the study variables or who never had any of the symptoms (see below), the sample was reduced to 16641 individuals. Ethical permission for the study was obtained from the ethics committee of the London School of Hygiene and Tropical Medicine, and the research was carried out in accordance with the Declaration of Helsinki.

### Study variables and statistical analysis

Information on the use of alternative (folk) medicine practitioners was obtained from a question which was designed to determine how respondents usually treated 10 symptoms (headache, chest pain, bad cough, breathlessness, unusual lump under the skin, warts, vomiting, fever, abdominal pain and diarrhoea) which are potentially severe enough to elicit a range of possible treatment choices. In relation to each one of the symptoms, respondents were asked ‘When you have [symptom X] how do you usually treat it?’ Using a ‘Yes’ and ‘No’ answer format, potential treatment options included ‘Go to a doctor or call the doctor or feldsher [medical assistant] at home’, ‘Go to a person practicing alternative (folk) medicine’, ‘Self-medicate with home-made medicines’, ‘Go to a pharmacist and buy medicines without a doctor’s prescription’, ‘Drink some alcohol’ ‘Do nothing’ ‘Other’ and ‘I have never had that symptom’. The question regarding the use of an alternative (folk) medicine practitioner was similar to a previous survey question that has been used to obtain information on the use of differing types of non-biomedical practitioner in contemporary Russia [18]. Respondents who answered ‘Yes’ to using an alternative (folk) medicine practitioner for any of the 10 symptoms were considered as users of these practitioners. The analyses were restricted to those who ever had at least one of the symptoms, as otherwise, they could not give meaningful answers about ‘usual treatment’. This excluded a small number of participants (1.2% of the sample). The country-wise prevalence of treatment by an alternative (folk) medicine practitioner was calculated as the number of those who ever went to one of these practitioners for any of the 10 symptoms from those who ever had at least one of those 10 symptoms.

Factors examined as potential determinants of alternative practitioner use included sex, age (categorised as 18–39, 40–64 and 65 and above), educational level (categorised as ‘high’ – complete and incomplete higher education – and ‘low’, everything below that level), marital status (married, single and divorced/widowed) and residential location – urban/rural. Socioeconomic status was assessed by asking respondents to evaluate their material living conditions in terms of one of four statements. Those who answered ‘yes’ to the statement that ‘the money is not enough even for our nutrition’ were categorised as having a ‘low’ level of material wealth. In contrast, respondents who had enough money to buy ‘long-lasting consumer goods’ or who stated that they did not have any material difficulties were categorised as having a ‘high’ level of wealth. A ‘middle’ category was assigned to those who had just enough money for nutrition and for articles ‘of the first level of material need’. Distrust of conventional medicine was assessed by asking respondents ‘To what extent do you personally trust doctors, nurses, other hospital staff?’ Answers were dichotomised into ‘quite/great trust’ scored ‘0’ and ‘rather/great distrust’ scored ‘1’. Respondents were also asked about the distance to their nearest health facility (doctor/feldsher/polyclinic). Answers were dichotomised into < 5 km and ≥ 5 km. Details of the study population are presented in Table 1. Those with missing values for age (0.01%), education (0.51%), marital status (0.59%), setting (0.82%), wealth (2.04%), and trust in doctors (5.12%) were excluded from all analyses. There were no missing values for sex and distance to the nearest health facility.

**Table 1:** Characteristics of the study population by country

**Categories**	**Armenia**	**Belarus**	**Georgia**	**Kazakhstan**	**Kyrgyzstan**	**Moldova**	**Russia**	**Ukraine**
	**(N=1867)**	**(N=1770)**	**(N=1664)**	**(N=1741)**	**(N=1877)**	**(N=1816)**	**(N=3735)**	**(N=2171)**
**Age**	%	%	%	%	%	%	%	%
18-39 years	39.5	38.1	34.2	50.0	54.8	36.2	38.6	33.0
40-64 years	41.4	42.2	48.1	39.1	34.5	45.8	43.4	43.1
≥65 years	19.1	19.7	17.7	11.0	10.7	18.1	18.1	23.9
**Sex**								
Male	40.3	44.4	43.0	44.5	45.5	45.0	43.3	38.6
**Education**								
High	25.8	19.5	40.0	25.9	26.8	19.4	25.7	24.6
**Marital status**								
Married	69.2	62.3	68.5	67.6	71.6	70.9	62.9	62.4
Single	13.9	14.0	14.8	15.8	15.1	8.2	13.8	10.9
Divorced/widowed	17.0	23.8	16.7	16.7	13.3	20.9	23.3	26.7
**Setting**								
Rural	38.9	30.7	41.9	47.4	59.8	58.0	27.2	32.0
**Wealth**								
Low	46.8	9.7	37.9	11.1	18.9	25.6	13.2	29.3
Middle	50.0	68.7	53.4	65.0	65.5	63.7	62.0	58.4
High	3.3	21.6	8.7	23.8	15.6	10.7	24.8	12.3
**Trust in doctors**								
Distrust	30.9	22.5	31.0	28.4	20.8	30.4	29.0	38.2
**Distance to health facility**								
≥5 km	10.8	8.8	6.6	5.6	10.7	1.9	5.9	9.2

Binomial logistic regression was used to assess which factors were associated with using an alternative (folk) medicine practitioner for each of the 10 symptoms. For all symptom-specific analyses only those who ever had that particular symptom were included. In the fully adjusted regression models the results are presented in the form of odds ratios (OR) with 95% confidence intervals (CI). Stata version 12.0 was used for the analysis (Stata Corp LP, College Station, Texas) and p<0.05 was set as the level of statistical significance.

## Results

**Figure 1:**
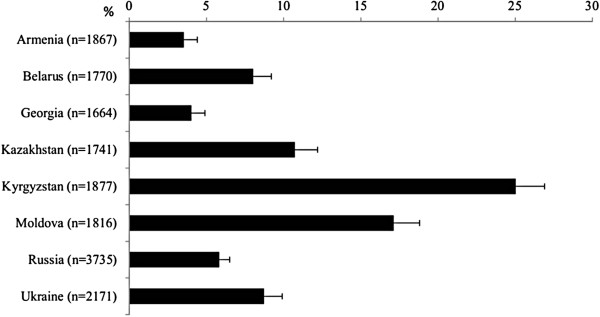
Prevalence of usual treatment by an alternative (folk) medicine practitioner among those who experienced at least one of ten common symptoms*.

In terms of the use of alternative (folk) medicine practitioners, in every country, respondents were most likely to consult them for warts, with the prevalence ranging from 10.8% in Armenia and Russia to 19.1% in Moldova (Table 2). The next most common reason for consulting was ‘an unusual lump under the skin’, with the figures ranging from 1.5% (Russia) to 11.0% (Moldova). Although in five countries fewer than 10% of respondents used alternative (folk) medicine practitioners to treat any of the symptoms, the figure was relatively high in others, ranging from 3.5% in Armenia to 17.1% in Moldova, and 25.0% in Kyrgyzstan (Figure 1).

**Table 2:** Percentage of those who usually go to an alternative (folk) medicine practitioner for treatment by symptom and country

Symptom*	**Armenia**	**Belarus**	**Georgia**	**Kazakhstan**	**Kyrgyzstan**	**Moldova**	**Russia**	**Ukraine**
Headache	0.7 (0.3-1.1)	0.5 (0.2-0.9)	0.3 (0.0-0.6)	1.3 (0.8-1.8)	3.0 (2.2-3.8)	1.6 (1.0-2.2)	0.8 (0.5-1.1)	0.9 (0.5-1.2)
Chest pain	0.5 (0.1-1.0)	0.8 (0.3-1.3)	1.5 (0.7-2.3)	2.7 (1.7-3.7)	3.4 (2.4-4.4)	4.8 (3.6-6.0)	0.9 (0.6-1.3)	1.6 (0.9-2.2)
Bad cough	1.0 (0.4-1.5)	1.5 (1.0-2.1)	1.8 (0.9-2.7)	1.3 (0.8-1.9)	5.1 (4.1-6.2)	5.5 (4.4-6.7)	0.9 (0.6-1.3)	1.8 (1.2-2.3)
Breathlessness	0.9 (0.1-1.6)	1.4 (0.5-2.3)	3.8 (1.8-5.8)	2.4 (1.1-3.6)	6.2 (4.8-7.6)	3.4 (2.0-4.9)	0.8 (0.3-1.3)	1.4 (0.7-2.1)
Unusual lump under the skin	1.9 (0.0-4.0)	3.6 (1.6-5.7)	2.8 (0.1-5.5)	8.5 (5.2-11.7)	5.2 (3.8-6.6)	11.0 (8.2-13.7)	1.5 (0.5-2.5)	3.0 (1.3-4.8)
Warts	10.8 (6.3-15.2)	14.2 (11.3-17.1)	11.0 (6.5-15.5)	18.8 (15.4-22.2)	18.7 (16.4-21.0)	19.1 (15.8-22.4)	10.8 (8.9-12.8)	15.0 (12.1-17.9)
Vomiting	1.0 (0.2-1.8)	0.8 (0.3-1.3)	0.6 (0.0-1.2)	0.8 (0.3-1.4)	6.8 (5.5-8.1)	3.6 (2.5-4.7)	1.0 (0.6-1.5)	1.2 (0.6-1.8)
Fever (≥3 days)	1.0 (0.4-1.5)	0.6 (0.2-0.9)	1.1 (0.5-1.7)	1.1 (0.5-1.6)	4.5 (3.5-5.5)	2.7 (1.9-3.6)	0.4 (0.2-0.7)	1.5 (1.0-2.1)
Abdominal pain	3.5 (2.2-4.8)	0.9 (0.4-1.5)	0.8 (0.2-1.4)	2.2 (1.2-3.1)	5.6 (4.5-6.8)	4.0 (2.9-5.1)	0.7 (0.4-1.1)	2.0 (1.3-2.7)
Diarrhoea	1.1 (0.5-1.8)	0.9 (0.4-1.4)	0.1 (0.0-0.3)	1.0 (0.5-1.5)	4.4 (3.4-5.4)	4.1 (2.9-5.2)	0.6 (0.3-0.9)	1.7 (1.1-2.3)

* Analysis is restricted to those who ever had the respective symptom.

Data is % (95% CI).

For all ten symptoms, respondents in Kyrgyzstan were significantly more likely to use an alternative (folk) medicine practitioner than their counterparts in Russia with odds ratios ranging from 1.7 (for warts) to 10.0 (fever ≥ 3 days), while the same was true for respondents in Moldova for 9 of the symptoms with odds ratios ranging from 1.8 (warts) to 6.4 (diarrhoea) (Table 3). Respondents were also significantly more likely to consult an alternative (folk) medicine practitioner in Kazakhstan and Ukraine when compared with Russia for at least 5 of the symptoms with the largest difference in odds being seen for an ‘unusual lump under the skin’ in Kazakhstan (OR: 5.80; CI: 2.55-13.22) and for ‘fever lasting 3 days or longer’ in Ukraine (OR: 3.52; CI: 1.80-6.87). For every symptom except a ‘bad cough’, rural residents were significantly more likely to use an alternative (folk) medicine practitioner than urban residents. There were no significant differences for any symptoms in terms of age or distance to the nearest medical facility. Having a ‘Middle’ or ‘High’ level of wealth was associated with a significantly higher likelihood of using these alternative practitioners compared to those with a low level of wealth for ‘chest pain’ (OR: 1.68; CI: 1.02-2.78 [High]), ‘bad cough’ (OR: 1.37; CI: 1.00-1.88 [Middle]), ‘abdominal pain’ (OR: 1.61; CI: 1.13-2.31 [Middle]) and ‘diarrhoea’ (OR: 1.72; CI: 1.14-2.61 [Middle]; OR: 1.75; CI: 1.03-2.99 [High]), while women were significantly more likely to consult this type of practitioner with warts (OR: 1.20; CI: 1.01-1.42) but less likely with ‘chest pain’ (OR: 0.68; CI: 0.51-0.91) and ‘fever ≥ 3 days’ (OR: 0.73; CI: 0.54-0.98). Educational differences were only observed for having an ‘unusual lump under the skin’ where respondents with a high education were significantly less likely to use an alternative (folk) medicine practitioner than those with low education (OR: 0.52; CI: 0.33-0.83). Finally, for two symptoms, vomiting (OR: 1.46; CI: 1.07-1.97) and diarrhoea (OR: 1.48; 1.10-1.98), those who distrust doctors were significantly more likely to use alternative (folk) medicine practitioners compared to those who trust doctors.

**Table 3:** Characteristics associated with obtaining treatment from an alternative (folk) medicine practitioner by symptom assessed by multivariate logistic regression

**Characteristic**	**Categories**	**Headache**	**Chest pain**	**Bad cough**	**Breathlessness**	**Unusual lump under the skin**	**Warts**	**Vomiting**	**Fever (**≥**3 days)**	**Abdominal pain**	**Diarrhoea**
		aOR (95%CI)*	aOR (95%CI)*	aOR (95%CI)*	aOR (95%CI)*	aOR (95%CI)*	aOR (95%CI)*	aOR (95%CI)*	aOR (95%CI)*	aOR (95%CI)*	aOR (95%CI)*
		N=16069	N=10385	N=13863	N=6292	N=3267	N=4621	N=9657	N=12874	N=10433	N=12425
Age (years)	18-39	1.00	1.00	1.00	1.00	1.00	1.00	1.00	1.00	1.00	1.00
	40-64	1.12 (0.80-1.58)	1.15 (0.82-1.60)	1.28 (0.98-1.69)	1.03 (0.70-1.51)	1.25 (0.87-1.80)	0.89 (0.74-1.07)	0.90 (0.65-1.25)	1.12 (0.80-1.55)	1.16 (0.85-1.57)	1.02 (0.74-1.40)
	≥65	0.84 (0.50-1.40)	0.90 (0.57-1.44)	1.09 (0.74-1.60)	0.76 (0.44-1.31)	0.81 (0.46-1.42)	0.76 (0.58-1.00)	1.17 (0.74-1.84)	0.99 (0.62-1.59)	1.27 (0.84-1.93)	0.80 (0.50-1.30)
Sex	Male	1.00	1.00	1.00	1.00	1.00	1.00	1.00	1.00	1.00	1.00
	Female	1.07 (0.79-1.46)	0.68 (0.51-0.91)^b^	0.88 (0.70-1.12)	0.87 (0.62-1.22)	1.14 (0.82-1.59)	1.20 (1.01-1.42)^a^	1.23 (0.92-1.66)	0.73 (0.54-0.98)^a^	1.04 (0.80-1.36)	1.16 (0.87-1.55)
Education	Low	1.00	1.00	1.00	1.00	1.00	1.00	1.00	1.00	1.00	1.00
	High	1.09 (0.76-1.57)	1.05 (0.74-1.49)	0.85 (0.64-1.14)	1.04 (0.70-1.55)	0.52 (0.33-0.83)^b^	1.04 (0.86-1.26)	1.00 (0.71-1.42)	0.91 (0.64-1.30)	1.17 (0.87-1.59)	0.73 (0.51-1.06)
Marital status	Married	1.00	1.00	1.00	1.00	1.00	1.00	1.00	1.00	1.00	1.00
	Single	0.74 (0.43-1.26)	0.97 (0.58-1.62)	1.18 (0.80-1.72)	1.11 (0.64-1.92)	0.53 (0.28-1.02)	0.78 (0.60-1.01)	1.04 (0.66-1.62)	0.84 (0.51-1.39)	1.24 (0.82-1.86)	0.71 (0.43-1.20)
	Divorced/widowed	1.14 (0.76-1.70)	1.40 (0.97-2.02)	1.33 (0.98-1.80)	1.40 (0.92-2.15)	0.95 (0.61-1.50)	0.86 (0.68-1.09)	1.11 (0.75-1.65)	1.51 (1.03-2.20)^a^	1.05 (0.73-1.50)	1.16 (0.80-1.70)
Setting	Urban	1.00	1.00	1.00	1.00	1.00	1.00	1.00	1.00	1.00	1.00
	Rural	1.76 (1.27-2.42)^b^	1.75 (1.29-2.37)^c^	1.22 (0.95-1.56)	1.94 (1.35-2.78)^c^	1.60 (1.11-2.29)^a^	1.33 (1.11-1.58)^b^	1.73 (1.26-2.36)^b^	1.58 (1.16-2.15)^b^	1.36 (1.03-1.79)^a^	1.43 (1.06-1.93)^a^
Wealth	Low	1.00	1.00	1.00	1.00	1.00	1.00	1.00	1.00	1.00	1.00
	Middle	0.88 (0.60-1.29)	1.31 (0.89-1.93)	1.37 (1.00-1.88)^a^	1.21 (0.78-1.87)	1.16 (0.73-1.82)	1.15 (0.91-1.45)	1.06 (0.73-1.55)	1.33 (0.90-1.96)	1.61 (1.13-2.31)^b^	1.72 (1.14-2.61)^a^
	High	0.98 (0.59-1.65)	1.68 (1.02-2.78)^a^	1.40 (0.91-2.13)	1.32 (0.74-2.36)	1.17 (0.66-2.10)	1.06 (0.79-1.42)	1.25 (0.76-2.05)	1.45 (0.86-2.44)	1.48 (0.91-2.42)	1.75 (1.03-2.99)^a^
Trust in doctors	Trust	1.00	1.00	1.00	1.00	1.00	1.00	1.00	1.00	1.00	1.00
	Distrust	1.33 (0.96-1.83)	1.04 (0.76-1.43)	1.19 (0.93-1.53)	1.19 (0.83-1.71)	1.41 (1.00-1.99)	1.16 (0.97-1.38)	1.46 (1.07-1.97)^a^	1.26 (0.92-1.72)	1.30 (0.98-1.72)	1.48 (1.10-1.98)^a^
Distance to health	<5	1.00	1.00	1.00	1.00	1.00	1.00	1.00	1.00	1.00	1.00
facility (km)	≥5	0.92 (0.53-1.61)	0.86 (0.47-1.56)	0.98 (0.63-1.54)	0.63 (0.32-1.27)	0.63 (0.30-1.33)	1.02 (0.75-1.40)	0.59 (0.31-1.10)	0.75 (0.42-1.34)	1.01 (0.63-1.60)	0.83 (0.48-1.43)
Country	Armenia	0.84 (0.43-1.65)	0.65 (0.24-1.73)	1.15 (0.57-2.32)	1.09 (0.37-3.19)	1.25 (0.32-4.85)	0.95 (0.56-1.59)	0.93 (0.37-2.34)	2.39 (1.03-5.54)^a^	5.31 (2.82-9.98)^c^	2.09 (0.97-4.50)
	Belarus	0.67 (0.32-1.42)	0.83 (0.38-1.81)	1.63 (0.97-2.75)	1.76 (0.74-4.17)	2.61 (1.05-6.47)^a^	1.37 (1.00-1.88)	0.77 (0.37-1.63)	1.27 (0.52-3.07)	1.26 (0.59-2.69)	1.44 (0.70-2.95)
	Georgia	0.37 (0.14-0.96)^a^	1.69 (0.85-3.36)	2.12 (1.14-3.91)^a^	4.50 (1.96-10.29)^c^	1.74 (0.51-5.92)	0.95 (0.57-1.56)	0.50 (0.15-1.68)	2.62 (1.20-5.72)^a^	1.07 (0.44-2.61)	0.19 (0.02-1.40)
	Kazakhstan	1.47 (0.83-2.58)	2.72 (1.54-4.81)^b^	1.41 (0.81-2.45)	2.54 (1.12-5.73)^a^	5.80 (2.55-13.22)^c^	1.72 (1.27-2.34)^b^	0.72 (0.33-1.56)	2.30 (1.08-4.87)^a^	2.92 (1.50-5.66)^b^	1.48 (0.73-3.01)
	Kyrgyzstan	3.37 (2.10-5.41)^c^	3.20 (1.89-5.43)^c^	5.85 (3.82-8.96)^c^	6.46 (3.31-12.62)^c^	3.27 (1.50-7.13)^b^	1.66 (1.26-2.17)^c^	6.33 (3.91-10.26)^c^	9.96 (5.40-18.36)^c^	7.88 (4.58-13.57)^c^	7.27 (4.25-12.43)^c^
	Moldova	1.66 (0.97-2.84)	4.52 (2.74-7.48)^c^	6.02 (3.95-9.20)^c^	3.53 (1.68-7.41)^b^	6.31 (2.92-13.62)^c^	1.75 (1.29-2.38)^c^	2.98 (1.75-5.08)^c^	5.61 (2.95-10.65)^c^	5.49 (3.13-9.65)^c^	6.38 (3.65-11.15)^c^
	Russia	1.00	1.00	1.00	1.00	1.00	1.00	1.00	1.00	1.00	1.00
	Ukraine	1.00 (0.55-1.82)	1.76 (0.98-3.16)	1.93 (1.19-3.15)^b^	1.68 (0.76-3.74)	2.01 (0.80-5.09)	1.41 (1.04-1.92)^a^	1.11 (0.59-2.10)	3.52 (1.80-6.87)^c^	2.73 (1.50-4.98)^b^	2.82 (1.56-5.10)^b^

* Mutually adjusted for all covariates in the model. Analysis is restricted to those who ever had the respective symptom; ^a ^P<0.05 ^b ^P<0.01 ^c^ P<0.001.

## Discussion

This study assessed the use of practitioners of alternative (folk) medicine for a range of common medical symptoms in eight fSU countries. To the best of our knowledge, this is the first study to compare the use of these practitioners in a number of fSU countries using a common research design and methodology, and also one of the few studies from Europe to look at the role of alternative practitioners in relation to a range of different medical symptoms. We found variation in the probability of using these practitioners among the countries, although for nearly all of the symptoms, rural residents were more likely to consult an alternative (folk) medicine practitioner than urban residents. There was also evidence that in some instances their use may be associated with greater wealth.

Before going on to discuss the main findings of this study, it is necessary to mention several possible limitations. Although our study sample was broadly representative of the population in each country, for some of the demographic variables, there were slight differences – such as the underrepresentation of men in Ukraine and Armenia and the overrepresentation of older persons (≥65 years) in Ukraine [26]. It is possible that these differences may have acted to bias some of our results, although this was at least partly addressed in the multivariate analysis. There may also have been problems relating to the main study variable used. The question enquiring about the use of alternative (folk) medicine practitioners did not refer to specific instances of treatment but rather ‘usual use’, and no specific time period was mentioned. More importantly, the question also failed to specify who exactly was providing the treatment. The use of the word ‘folk’ in parentheses in the question may have led some respondents to interpret the question as referring solely to folk medicine practitioners while others may have interpreted the term more broadly to include practitioners of alternative medicine more generally where ‘folk’ medicine is merely one example of alternative medicine. Given this potential for uncertainty, one reviewer suggested that it would be preferable if the exact question answer option was used throughout the text. Although we have done this, it does not solve the problem of the question possibly having been interpreted in different ways by different respondents. In addition, medical doctors can (and sometimes do) provide folk or other alternative treatments in these countries [24,32] and it is possible that respondents could have interpreted the question more generally as referring to any type of practitioner providing these treatments. The ability to generalise our findings might be further limited by the fact that the understanding of what constitutes alternative (folk) medicine may vary between the countries as well as by the wide diversity of alternative treatments available in this region [32] which may also vary between locations. Finally, we cannot exclude the possibility that important variables may have been missing from the analysis as we had no information on such things as ‘lay referral systems’ i.e. social networks [19] which have been linked to the use of non-biomedical practitioners in some of the countries in this region previously.

The current study revealed large differences in the use of alternative (folk) medicine practitioners in eight former Soviet countries with the prevalence ranging from 3.5% (Armenia) to 25.0% (Kyrgyzstan). It is possible that these differences might be related to the different government policies on complementary and alternative medicine. Three of our study countries (Kazakhstan, Kyrgyzstan and Ukraine) have explicit policies on the training and licensing of alternative practitioners (with Russia regulating the alternative medicine sector) [14], while the country with seemingly the strictest policy – Kyrgyzstan – where ‘healers’ must be medically trained [14,30], has by far the greatest prevalence of alternative practitioner use (although this may simply reflect an attempt to take control of a practice that was already widespread).

In terms of the factors associated with the use of alternative (folk) medicine practitioners for the treatment of different symptoms, rural residents were significantly more likely to use these practitioners for nearly every symptom than urban residents. This might be driven by poorer access to conventional medical services and dissatisfaction with the provision of mainstream health care [33] which have been connected with the greater use of alternative forms of medical care in other rural contexts [34]. This said, in the current study, we found that access (as modelled by distance to the nearest health service provider) was not associated with differences in use – despite the sometimes critical shortage of rural health personnel in countries such as Kyrgyzstan [30]. While it is possible that this variable failed to capture other problems encountered by rural residents such as the poor medical provisioning of existing rural health facilities [30], it could also indicate that other social and cultural factors are important e.g. that rural residents are simply continuing to visit those service providers which they had access to and used, albeit covertly, in the Soviet period.

Another factor which could underpin the use of alternative (folk) practitioners throughout this region is their cost. Against a backdrop of growing impoverishment where out-of-pocket payments for conventional medical care are now widespread and where ‘unaffordability’ is the major reason for forgoing needed treatment in some of our study countries [26], complementary medicine [35] and traditional healers [36] may be comparatively cheap – possibly linked to the tradition in this region that users themselves should determine the exact form of reimbursement [19]. However, the results from the current study seem to contradict this notion as possessing greater wealth was associated with a significantly higher likelihood of using a non-biomedical practitioner for almost half of the symptoms. This finding supports earlier research from Russia which has suggested that the use of alternative medicine may not be necessarily cheaper than conventional treatments [18] and that ‘there is a strong positive relationship between socioeconomic status and [the] aggressive pursuit of health care options – both within and outside of the traditional medical system’ [19]. Given this, it might be the case that using a practitioner of alternative (folk) medicine is indicative of a ‘wealth divide’ with those who are poorer perhaps relying on self-treatment with traditional remedies as a form of coping strategy in an environment where conventional health service use is increasingly costly.

Our results also indicate that distrust of conventional medicine may be associated with the use of alternative practitioners for some symptoms – a finding which mirrors that from other locations where a lack of confidence in medical doctors has also been linked to the use of folk medicine [37]. Earlier research from the countries in this region has highlighted how a lack of trust in the qualifications of medical staff is associated with refusing necessary medical care [26]. This distrust might have resulted from having experienced negative medical outcomes from conventional treatments, which are seemingly commonplace [19], although the idea that the fear of such outcomes is a principal motivation in the use of alternative practitioners has been rejected by some authors [19]. Nonetheless, negative interactions with doctors (which can underpin both distrust and the refusal of conventional medical treatment) are widespread in this region [19] and have been listed as a factor spurring the use of non-biomedical practitioners in one fSU country [28].

## Conclusions

By examining a variety of symptoms we have been able to highlight how a complex range of factors underlie the use of practitioners of alternative (folk) medicine in the countries of the fSU. While rural residence and wealth seem to be important in relation to a range of symptoms, some characteristics such as distrust of doctors were associated with the use of these practitioners for fewer symptoms, while for other factors such as age, no association was observed despite previous research from other countries suggesting that it is important [38,39]. The finding that the use of alternative (folk) medicine is widespread in some fSU countries, in conditions where there has been a sharp growth in the number of unlicensed and unregulated practitioners in some of these countries in the post-Soviet period [28], highlights the need for more country-specific research. Moreover, the fact that the use of alternative (folk) medicine practitioners is likely to be merely the ‘tip of the iceberg’ in terms of the use of complementary and alternative medicine in the countries in this region indicates the urgency of this task and of the need for future research to focus on both the use of practitioners *and* self-treatment in order to better understand CAM, what factors are associated with its use and how it is impacting on population health.

## Abbreviations

fSU: Former Soviet Union; LLH: Living Conditions, Lifestyles and Health survey.

## Competing interests

The authors declare that they have no competing interests.

## Authors’ contributions

AS conceived the idea for the study and wrote the body of the text. AK analysed the data and reviewed and commented on the manuscript for intellectual context. ER, BR, DB and MM all critically reviewed the manuscript and added parts to the text. The final version of the manuscript was approved by all authors.

## Pre-publication history

The pre-publication history for this paper can be accessed here:

http://www.biomedcentral.com/1472-6882/13/83/prepub
